# Editorial—Hub Me

**Published:** 1893-12

**Authors:** 


### EDITORIAL OUTLOOK.

### HUB ME.

Passing along Broadway some time ago, the vehicle was arrested by some slight obstruction and the horses were not quite able to start it; the driver saw at once that but a very little aid was needed, and, turning to another Jehu who was coming behind him, said, "*Hub me, shipmate.*" The other saw as instantly what was required and without a moment's hesitation so guided his own horses as to make the hub of his own wagon strike lightly against that of the other, and each giving his own animals a touch of the whip, both vehicles moved on almost as easily as if nothing had happened.

How many times in the great Broadway of life might men "hub" one another without incommoding themselves! A friendly act done, an obligation incurred, some future act of kindness provoked, at the expense of a word or only a single moment's time, The most of us regard truck and express drivers as rather rough specimens of humanity; but ever since the incident just related, we have seen a moral beauty in the odd expression, "Hub me, Shipmate." When a man takes a newspaper or a periodical, he usually becomes attached to it, begins to feel that its editor is his friend; and as often as the publication comes, he derives from the work of its editor some interesting item of news, some amusing statement or some profitable idea or suggestion. This is repeated a dozen, fifty or hundreds of times a year, for which the dollar or two, or five of subscription price is not the shadow of a compensation singly. Under the circumstances, then, we appeal to each reader of this editorial in behalf of any publication which he receives, to help it to a new subscriber, as often as an opportunity is afforded, by a single word of approbation or solicitation. There are many persons who have so much of the milk of human kindness in them, that they would take a paper rather than refuse; and for that courtesy you have chances of doing them a service just in proportion to the real worth of the publication commended. To each present subscriber of our Journal we venture the appeal, with some confidence.

"Hub me, Shipmate."

We are to have a limited number of the 1893 Journals handsomely bound in cloth binding, which we offer at the low price one dollar and forty cents each. Send in your orders at once.

